# Faltering of prenatal growth precedes the development of atopic eczema in infancy: cohort study

**DOI:** 10.2147/CLEP.S175878

**Published:** 2018-12-12

**Authors:** Sarah El-Heis, Sarah R Crozier, Eugene Healy, Sian M Robinson, Nicholas C Harvey, Cyrus Cooper, Hazel M Inskip, Janis Baird, Keith M Godfrey, On behalf of Southampton Women’s Survey Study Group

**Affiliations:** 1 Medical Research Council Lifecourse Epidemiology Unit, University of Southampton, Southampton, UK, kmg@mrc.soton.ac.uk; 2 Dermatopharmacology, Faculty of Medicine, University of Southampton, Southampton, UK; 3 NIHR Southampton Biomedical Research Centre, University of Southampton and University Hospital Southampton NHS Foundation Trust, Southampton, UK, kmg@mrc.soton.ac.uk; 4 NIHR Musculoskeletal Biomedical Research Unit, University of Oxford, Oxford, UK; 5 Institute of Developmental Sciences, University of Southampton, Southampton, UK, kmg@mrc.soton.ac.uk

**Keywords:** atopic eczema, fetal growth, infant growth

## Abstract

**Background:**

Infants with atopic eczema have an increased risk of impaired growth, but the origin of this impairment is unclear. The aim of this study was to examine fetal and infant growth in relation to infantile atopic eczema.

**Methods:**

Within the UK Southampton Women’s Survey, 1,759 infants with known maternal menstrual data had anthropometric measurements at 11, 19, and 34 weeks’ gestation, birth, and ages 6 and 12 months, enabling derivation of growth velocity SD scores. Infantile atopic eczema at ages 6 and/or 12 months was ascertained using modified UK Working Party diagnostic criteria.

**Results:**

Expressed per SD increase, higher femur length and abdominal circumference at 34 weeks’ gestation were associated with decreased risks of atopic eczema (eczema OR/SD increase 0.81 [95% CI 0.69–0.96], *P*=0.017 and 0.78 [95% CI 0.65–0.93], *P*=0.006, respectively), while every SD increase in head to abdominal circumference ratio (indicating disproportionate growth) was associated with an increase in risk of atopic eczema (1.37 [1.15–1.63], *P*=0.001). Lower velocities of linear growth from 11 weeks’ gestation to birth and birth to age 6 months were associated with atopic eczema (atopic eczema OR/SD increase 0.80 [0.65–0.98], *P*=0.034 and 0.8 [1 0.66–1.00], *P*=0.051, respectively). Infants with atopic eczema at age 12 months had a larger head circumference in early gestation and faltering of abdominal growth velocity from 19 to 34 weeks’ gestation (atopic eczema OR/SD increase 0.67 [0.51–0.88], *P*=0.003).

**Conclusion:**

Infants with atopic eczema demonstrate altered patterns of fetal growth, including faltering of linear growth in utero, prior to the clinical onset of atopic eczema. These findings suggest growth falters prior to the start of clinical atopic eczema and its treatment.

## Introduction

Linear growth impairment in children with atopic eczema is a clinical concern.^1^–^3^ National recommendations in USA,^4^ UK,^5^,^6^ and other settings are that growth is monitored as part of clinical care for children with atopic eczema. Possible reasons for growth faltering have been proposed and include effects of the inflammatory disease,^7^ corticosteroid treatment,^8^ poor nutrition as a result of an inappropriately restrictive diet,^9^ and eczema associated sleep disturbance.^10^ Hitherto, little attention has been paid to the possibility of premorbid changes in growth trajectory in infants with atopic eczema. Any such changes in premorbid growth may help explain the growth impairment in children with atopic eczema while also providing insights into etiology of the skin disorder. The development of inflammatory diseases such as atopic eczema is influenced by both genetic determinants and environmental exposures in early life, including poor nutrition,^11^,^12^ maternal stress,^13^,^14^ smoking,^15^ and microbiome-related exposures.^16^ With increasing evidence that atopic disease is partly determined by the fetal environment, better understanding of early life environment becomes crucial for identifying potential preventative strategies.

Studies examining infant birthweight in relation to atopic eczema have been inconsistent,^17^,^18^ and it is now recognized that infant birthweight is only a crude proxy for patterns of fetal growth, which may show stronger associations with later outcomes than cross-sectional assessments of size at single time points.^19^ A previous study reported that fetuses with below average crown-rump length at 11 weeks gestation and an above average biparietal diameter at 19 weeks’ gestation were more likely to have eczema ascertained by postal questionnaire at age 10 years,^20^ but no previous study has examined longitudinal measures of fetal and infant size in relation to infantile atopic eczema. More information is available for other atopic outcomes, including evidence that a large neonatal head circumference and higher abdominal circumference growth velocity between 11 and 19 weeks’ gestation have been linked to an increased risk of atopy.^19^

In this study, we examined fetal and infant anthropometric measurements and growth velocities in relation to the risk of atopic eczema at ages 6 and 12 months, to look for evidence of altered growth prior to the clinical onset of atopic eczema which might support a prenatal developmental influence on the disorder.

## Methods

### Southampton Women’s Survey (SWS)

In the UK SWS, between 1998 and 2002, 12,583 women aged 20–34 years who were not pregnant were recruited from the general population through general practitioners in Southampton, UK. Information on maternal diet, lifestyle, socioeconomic status, and body composition was collected.^21^ Women who became pregnant were followed up through their pregnancies; ultrasound measurements of fetal size were performed at 11, 19, and 34 weeks. A total of 3,158 live-born singleton infants were delivered. Further anthropometry was performed at birth and at ages 6 and 12 months. The findings reported here are based on 1,759 term, live singleton births with no congenital abnormalities, who were assessed for atopic eczema at 6 and/or 12 months and had fetal and infant anthropometric measurements and known maternal menstrual data. Neonatal deaths and infants with major congenital anomalies, gestational age <37 weeks, and missing atopic eczema data at 6 and/or 12 months were excluded from the analyses (Figure S1). All phases of the SWS were approved by the Southampton and South West Hampshire Local Research Ethics Committee, and parents gave written informed consent.

### Outcome assessment

Case definition of atopic eczema was based on the UK Working Party diagnostic criteria for the definition of atopic eczema,^22^ using information collected by trained research nurses who administered a standard questionnaire and ascertained other information required for the diagnostic criteria (a combination of history of itchy skin condition and two or more of the following: history of involvement of the skin creases such as folds of elbows, behind the knees, fronts of ankles, cheeks, or around the neck; a history of a general dry skin in the last year; and visible flexural eczema or eczema involving the cheeks/forehead and outer limbs). All infants were assessed for atopic eczema before the age of 2 years, thus this UK Working Party criterion was met by all infants in the study cohort. However, as the infants were not old enough to have developed clearly defined atopic disorders, a personal history of atopy was omitted as a criterion.

### Fetal and infant anthropometric measurements

At 11, 19, and 34 weeks’ gestation, women underwent high-resolution ultrasound scanning by experienced research staff using Kretz Voluson^®^ 730 (GE Kretz Ultrasound, Tiefenbach, Austria) or Acuson Sequoia^®^ 512 (Siemens, Erlangen, Germany) systems, which was cross-calibrated. Measurements of fetal linear size (crown-rump length at 11 weeks, femur length at 19 and 34 weeks), head circumference (at 11, 19, and 34 weeks), and abdominal circumference (at 11, 19, and 34 weeks) were made according to an internationally accepted and validated methodology.^23^ Postnatal anthropometry was performed by trained research nurses according to standardized procedures, with each measurement repeated three times and the mean value used for analysis. Crown-heel length at birth was measured using a neonatometer (Harpenden, Wrexham, UK) and at ages 6 and 12 months using an infantometer (Seca Ltd, Birmingham, UK); head and abdominal circumferences were measured using unmarked tapes read off against a metal ruler at birth, 6, and 12 months.

### Statistical analyses

Summary statistics are presented as mean (SD) or median (IQR) for continuous variables, and percentages for categorical variables.

As measurements were taken close to but not at the exact ages specified, the associations between anthropometric measures and age were modeled using Cole’s LMS^24^ in LMSchartmaker,^25^ to create sex-specific size-for-age *z*-scores. This method provides smooth centile curves to potentially skewed data, enabling calculation of *z*-scores at exact ages. The method summarizes the changing distribution of the anthropometric parameters by age using three curves representing the skewness (L), the median (M), and the coefficient variation (S). These parameters are all used together to create the preferred percentiles. LMS has been adopted in many studies and is considered a reliable method of smoothing growth curves.^26^–^29^

Conditional models of change were built using linear regression analysis: thus size *z*-score at 11 weeks was the starting point. Conditional change in *z*-score from 11 to 19 weeks was defined as the standardized residuals resulting from the linear regression model of *z*-score at 19 weeks on *z*-score at 11 weeks. The conditional change in *z*-score from 19 to 34 weeks was obtained from the standardized residuals resulting from regressing *z*-score at 34 weeks on both *z*-score at 19 weeks and *z*-score at 11 weeks simultaneously. This process was continued for each subsequent time point, resulting in independent measures of conditional growth. Using the measures of linear size at 11 weeks, birth, and 6 months, additional conditional growth measures using this subset of time points were created: linear size at 11 weeks, linear growth from 11 weeks to birth, from birth to 6 months, and from 6 to 12 months. Measures of conditional growth were mutually uncorrelated and yielded SD scores, enabling comparison of relationships between growth in different time intervals and risk of atopic eczema at ages 6 and 12 months.

Potential confounding variables were determined prior to the analysis using a directed acyclic graph (DAG) (Figure S2). DAGs provide a robust and objective means of selecting confounders. DAGs are a graphical representation of causal effects between variables. Based on prior knowledge and usual convention for the causal effects studied, these graphs identify potential confounding variables which are then adjusted for multivariate analysis to minimize confounding bias.^30^ Additionally, the graphs identify competing exposures that can be adjusted for to improve the precision of the model. The resulting factors that were included as confounding variables in our analyses were maternal body mass index (BMI) at initial assessment, educational attainment (maternal age and parity were also considered, but the DAG indicated their inclusion was not appropriate), infant gestational age, and breastfeeding duration. Competing exposures identified and adjusted for were maternal eczema in the 12 months prior to the initial assessment, maternal smoking, and infant sex. *P*<0.05 was considered statistically significant. Logistic regression analyses were performed (Stata version 14.1; StataCorp LP, College Station, TX, USA) to relate fetal and infant anthropometric measures (in SD) and growth velocities (in SD) to infant atopic eczema at ages 6 and 12 months, with results presented as atopic eczema OR per SD increase (OR/SD).

## Results

### Cohort characteristics

Table 1 summarizes maternal, fetal, and infant characteristics. Among the study group, the mothers’ mean (SD) age at their child’s birth was 31.0 (3.7) years, 52.2% were primiparous, 11.7% smoked during pregnancy, 6.7% of mothers had eczema in the past 12 months, 51.1% of infants were male, mean (SD) infant birthweight was 3.52 (0.47) kg, and median gestational age at birth was 40.1 weeks (IQR 39.3–41.0). A total of 1,698 infants were assessed for atopic eczema at age 6 months, 9.5% of them had atopic eczema. At age 12 months, 1,684 infants were assessed and 10.0% had atopic eczema. Table S1 shows the characteristics of the 1,759 participants in the study group in comparison with the overall SWS pregnancy cohort; the study group mothers were slightly older at child’s birth, higher proportions were primiparous or had attained A level or higher education, and smoking was less prevalent.

**Figure 1:**
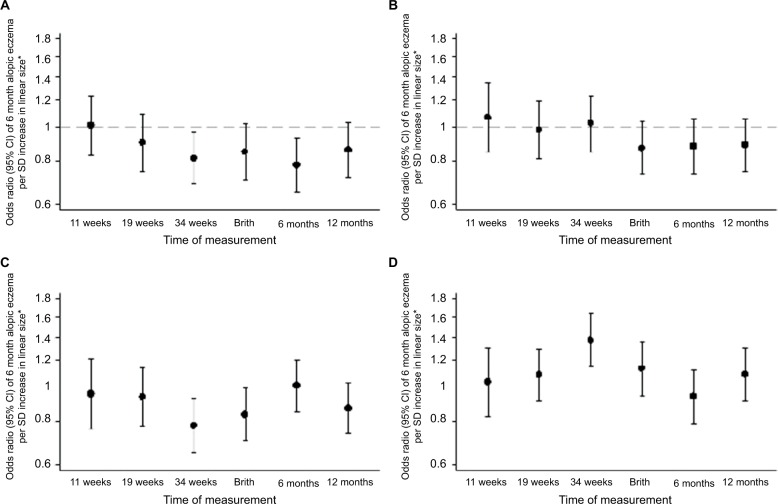
Size measurements in relation to atopic eczema at age 6 months. **Notes: (A)** Linear size, **(B)** head circumference, **(C)** abdominal circumference, and **(D)** head:abdominal circumference ratio. *Controlling for gestation, sex, breastfeeding, maternal BMI, qualification, maternal eczema, and smoking in pregnancy.

**Figure 2:**
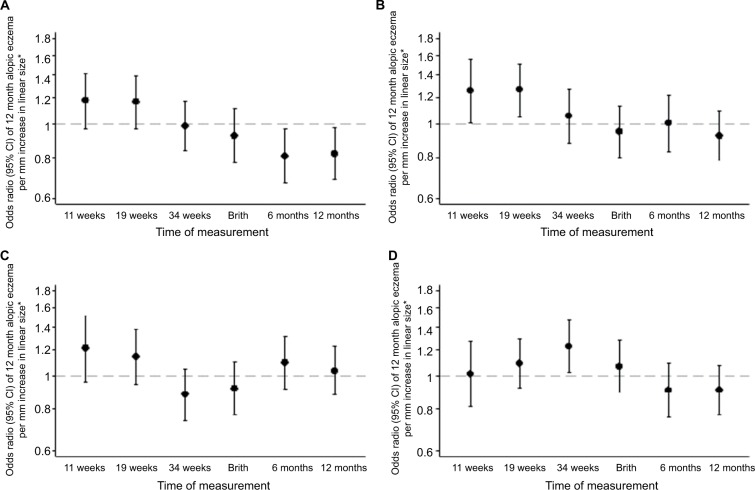
Size measurements in relation to atopic eczema at age 12 months. **Note: (A)** Linear size, **(B)** head circumference, **(C)** abdominal circumference, and **(D)** head:abdominal circumference ratio. *Controlling for gestation, sex, breastfeeding, maternal BMI, qualification, maternal eczema, and smoking in pregnancy.

Univariate (unadjusted) and multivariate (adjusted) analyses of atopic eczema at ages 6 and 12 months in relation to fetal and infant size measurements are shown in Table S2. Figures 1 and 2 show the ORs of atopic eczema in relation to fetal and infant size measurements at ages 6 and 12 months, respectively. Postnatal anthropometry showed that infants with atopic eczema at 6 months were shorter at age 6 months (eczema OR/SD increase 0.78, 95% CI 0.65–0.93, *P*=0.006) and that those with atopic eczema at 12 months were shorter at ages 6 and 12 months (eczema OR/SD increase 0.81, 95% CI 0.67–0.97, *P*=0.021 and OR 0.82, 95% CI 0.69–0.98, *P*=0.028, respectively).

### Associations of fetal size and growth velocities with infant atopic eczema at age 6 months

At 34 weeks’ gestation, a shorter femur length, smaller abdominal circumference, and higher head to abdominal circumference ratio were associated with increased risks of atopic eczema at age 6 months. Expressed per SD increase, higher femur length and abdominal circumference were associated with decreased risks of atopic eczema (eczema OR/SD increase 0.81, 95% CI 0.69–0.96, *P*=0.017 and 0.78, 95% CI 0.65–0.93, *P*=0.006, respectively), while every SD increase in head to abdominal circumference ratio (indicating disproportionate growth) was associated with an increase in risk of atopic eczema (1.37, 95% CI 1.15–1.63, *P*=0.001) (Figure 1). Fetal head circumference was not related to infant atopic eczema at age 6 months (Figure 1).

**Figure 3:**
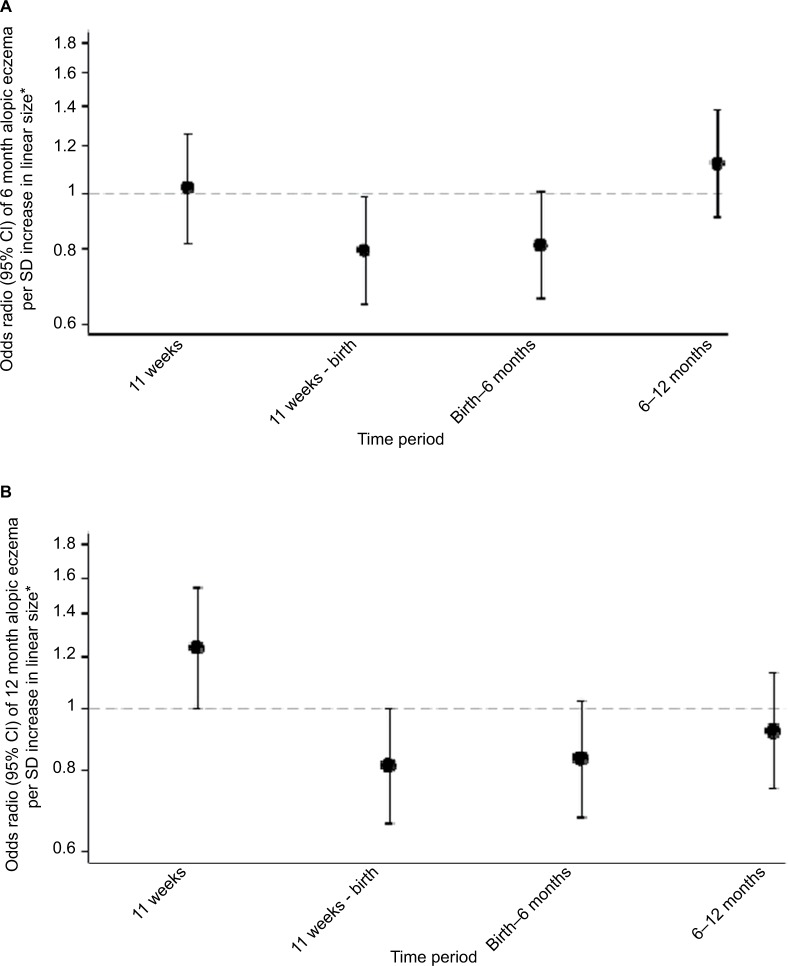
Linear growth velocities in relation to atopic eczema at ages 6 and 12 months. **Notes:** *Controlling for gestation, sex, breastfeeding, maternal BMI, qualification, maternal eczema, and smoking in pregnancy. (**A**) Linear growth velocities in relation to atopic eczema at age 6 months. (**B**) Linear growth velocities in relation to atopic eczema at age 12 months. **Abbreviations:** CRL, crown-rump length; FL, femur length; CHL, crown-heel length.

A lower velocity of linear growth from 11 weeks’ gestation to birth was associated with atopic eczema at age 6 months (atopic eczema OR/SD increase 0.80, 95% CI 0.65–0.98, *P*=0.034) (Figure 3A); this particularly reflected lower linear growth velocity from 11 to 19 weeks’ gestation (Table S3). Lower velocity of linear growth from birth to 6 months also showed a trend toward significance (atopic eczema OR/SD increase 0.81, 95% CI 0.66–1.00, *P*=0.051). A lower abdominal circumference growth velocity from 19 to 34 weeks’ gestation was associated with an increased risk of atopic eczema at age 6 months (eczema OR/SD increase 0.71, 95% CI 0.55–0.92, *P*=0.009), but there were no associations with head circumference growth velocities (Table S3).

### Associations of fetal size and growth velocities with infant atopic eczema at age 12 months

Infants with atopic eczema at age 12 months had a larger head circumference in early pregnancy (at 11 and 19 weeks’ gestation eczema OR/SD increase 1.25, 95% CI 1.00–1.56, *P*=0.045 and 1.26, 95% CI 1.05–1.51, *P*=0.012, respectively), faltering of abdominal circumference growth velocity from 19 to 34 weeks’ gestation (eczema OR/SD increase 0.67 [0.51–0.88], *P*=0.003) and a higher head to abdominal circumference ratio at 34 weeks (eczema OR/SD increase 1.22, 95% CI 1.03–1.46, *P*=0.025), with trends toward faltering of linear growth velocity from 11 weeks to birth and birth to age 6 months (eczema OR/SD increase 0.81 [0.66–1.00], *P*=0.051 and 0.83 [0.68–1.03], *P*=0.087, respectively) (Figure 3B and Table S3).

## Discussion

We found that infants with atopic eczema at age 6 months have faltering of linear growth beginning after 11 weeks’ gestation, with a higher head to abdominal circumference ratio at 34 weeks’ gestation. Infants with atopic eczema at age 12 months had a larger head circumference in early pregnancy and faltering of abdominal growth in the second half of pregnancy. Postnatally, the infants with atopic eczema at ages 6 and 12 months were shorter than infants without atopic eczema, but the longitudinal measurements of fetal size suggest that this growth faltering commenced prior to birth. These associations were robust to adjustment for potentially confounding variables, notably maternal age, BMI, education, smoking in pregnancy, and eczema in the 12 months prior to the initial assessment at preconception and infant sex, and duration of breastfeeding.

Our study presents the first longitudinal data examining fetal and infant growth velocities to infant atopic eczema at ages 6 and 12 months. Previous studies have generally focused on anthropometric measurements at birth or during infancy, and have found that they were not related to the prevalences of reported eczema or hay fever by the age of 13 years,^31^ or to eczema at age 7 years.^32^ The same infant size at birth can be achieved through different patterns of fetal growth, and few previous studies have examined patterns of fetal growth in relation to atopic outcomes. An increase in size between first trimester crown-rump length and second trimester biparietal diameter has been associated with higher risks of eczema and asthma at age 10 years.^20^ Pike et al^19^ reported that rapid early gestation fetal abdominal growth followed by late gestation faltering of abdominal circumference growth was associated with later atopy at age 3 years, and late gestation abdominal growth faltering with atopic wheeze.

Mechanistically, it is known that intrauterine growth restriction leads to disproportionate fetal growth and a high head to abdominal circumference ratio as a result of “brain sparing” responses, which direct nutrient-rich blood to maintain brain growth away from truncal organs including the thymus, with potential impact on immune development. Animal and human studies have linked fetal and birth anthropometric parameters indicative of undernutrition during pregnancy with smaller thymic size and impaired thymic development.^12^,^33^,^34^ We found that a larger fetal head circumference at 11 and 19 weeks’ gestation was linked with a higher risk of atopic eczema, with evidence of disproportionate head to abdominal circumference at 34 weeks. Therefore, we suspect that these growth alterations might influence thymus development, resulting in a diminished population of T helper (Th) 1 lymphocytes, favoring Th2 populations and consequently raised serum IgE,^35^ an immune reaction that is seen in atopic eczema and other atopic conditions.^36^ However, the exact mechanisms responsible for the association of altered fetal growth with development of atopic eczema are unknown, the observation points to involvement of periconception or early pregnancy factors in the etiology of infantile atopic eczema. The patterns of association between anthropometric measurements and infant atopic eczema at ages 6 and 12 months differed; this could reflect a chance finding or heterogeneity in the etiology and pathogenesis of atopic eczema in early childhood.^37^

Hitherto, it has been thought most likely that the chronic inflammatory process associated with atopic eczema results in growth impairment as proinflammatory cytokines such as IL-6, which promotes Th2 differentiation and simultaneously inhibits Th1, can act at the level of the growth plate, or may alter the growth hormone insulin-like growth factor 1 (IGF-1) axis.^7^ Animal studies show growth impairment in juvenile chronic arthritis and chronic inflammatory bowel disease independent of nutrition as a result of an IL-6-mediated decrease in IGF-1.^38^,^39^ Alternatively, it has been proposed that infants with atopic eczema may be susceptible to postnatal growth impairment due to treatment of the condition with topical or systemic corticosteroids,^8^ poor nutrition as a result of an inappropriately restrictive diet,^9^ and associated disturbance in sleep.^10^ However, our findings suggest that intrinsic and intrauterine factors that influence growth may modify the risk of developing atopic eczema as opposed to growth faltering developing postnatally as a result of the inflammatory skin condition or its treatment. These findings have important clinical implications and suggest that improved control of the inflammatory process in infantile atopic eczema or avoidance of topical corticosteroids may not necessarily resolve the growth impairment seen in many infants with atopic eczema.

Strengths of this study are its large sample size, its prospective nature, and the standardized assessment of fetal/infant size and atopic eczema by trained staff. Only a sub-sample of the SWS cohort was studied as calculation of fetal growth velocities in early gestation requires secure menstrual information; otherwise the estimated date of conception has to be “set” from a measurement of fetal size in the first half of pregnancy, thereby negating the scope for examination of early gestation effects. The mother–offspring pairs included in the study were therefore those with known menstrual data information and consistent ultrasound data, allowing for accurate size-for-age fetal and infant measurements and calculation of growth velocities. Mothers in the study group were slightly older at child’s birth, higher proportions were primiparous or had attained A-level or higher education, and smoking was less prevalent when compared with the overall SWS pregnancy cohort. These factors were considered in the DAG, which indicated that maternal education and smoking during pregnancy were potential confounding variables and were therefore corrected for the statistical analysis. Numerous potentially confounding factors were considered, and the DAG identified those that should be included in the statistical analyses. This objective method provides a robust means of assessing the causal relationship between exposure and outcome. Residual confounding cannot, however, be completely excluded. Limitations of the study include the use of questionnaire-based assessments for part of the assessment for the diagnosis of atopic eczema, which may introduce bias; however, the assessment also involved a clinical examination undertaken by trained staff. The UK Working Party Diagnostic Criteria for Diagnosis of Atopic Dermatitis are highly sensitive and specific for identifying cases of atopic eczema, particularly if applied to developed countries;^40^–^44^ although they do not assess the severity of the disease, they represent the most comprehensively validated criteria for the diagnosis of atopic eczema, in both community and hospital settings.^45^,^46^ The criteria were modified to omit atopic disease in a first-degree relative from our case definition to avoid too narrow focus on familial cases of atopic eczema and to prevent excluding an important group of infants from such studies as we were seeking to disentangle the apparent heterogeneous phenotypes that “atopic eczema” is now thought to represent.^34^ Maternal history of atopic eczema in the 12 months prior to the initial assessment at preconception, however, was considered as a confounding variable as determined by the DAG (Figure S2). Severity of atopic eczema was not assessed, and it may be possible that those with clinically mild atopic eczema may not exhibit the same growth impairment as those with a more severe condition.^10^ Although exploratory and hypothesis-generating methods were used to determine the described growth patterns and multiple statistical testing is a potential limitation of this study, only three parameters (linear size, abdominal circumference, and head circumference) were examined to lessen the inherent risks.

## Conclusion

Our study demonstrates links between fetal and infant anthropometric measurements and growth patterns with risk of atopic eczema at ages 6 and 12 months. The findings suggest that growth falters prior to the onset of the inflammatory process associated with atopic eczema or its treatment and provide additional support for important prenatal influences on this skin condition.

## Supplementary materials

Figure S1 Selection of study group sample from the Southampton Women’s Survey (SWS) cohort.
**Note:** *Non assisted conception, regular cycle, sure/certain of last menstrual period (LMP), not on oral contraceptive pill prior to LMP, dating range scan data available, LMP consistent with date of conception, first positive pregnancy test, scan data, and gestation at birth.

Figure S2 Fetal and infant growth and atopic eczema DAG.
**Notes:** Confounding variables: maternal BMI, maternal education, gestational age, and breastfeeding duration. Competing exposures (variables adjusted for to improve precision of model): maternal eczema, smoking during pregnancy, and infant sex.
**Abbreviations:** BMI, body mass index; DAG, directed acyclic graph.

**Table S1:** Comparison of the study population with the remainder of the SWS participants

Characteristics	Study population (n=1,759)	Other SWS participants * (n=1,169)	*P*-value for difference between the two groups

**Maternal**			
Age at child’s birth (years)	31.0 (3.7)	30.2 (4.0)	<0.001
A level or higher degree (%)	63.3	52.6	<0.001
Smoking in pregnancy (%)	11.7	22.3	<0.001
Primiparous (%)	52.2	47.8	0.021
Eczema in the last 12 months (%)	6.7	7.3	0.56
Prepregnancy BMI (kg/cm ^2^)	24.1 (21.9–27.4)	24.2 (21.9–27.3)	0.86
**Infant**			
Male (%)	51.1	52.0	0.65
Gestational age (weeks)	40.1 (39.3–41.0)	40.2 (39.2–41.0)	0.71
Birth weight (kg)	3.52 (0.47)	3.49 (0.47)	0.08

**Notes:**

* Includes live singleton infants born at term, with no congenital abnormalities and who survived the neonatal period. Values indicate median (IQR), mean (SD), or %.

**Abbreviations:** SWS, Southampton Women’s Survey; BMI, body mass index.

**Table S2:** Static size measurements in relation to eczema at ages 6 and 12 months

Size measurements	Eczema at age 6 months								Eczema at age 12 months							
	Univariate				Multivariate *				Univariate				Multivariate *			
	n	OR	95% CI	*P*-value	n	OR	95% CI	*P*-value	n	OR	95% CI	*P*-value	n	OR	95% CI	*P*-value

**Linear size (SD)**																
11 weeks (CRL)	1,434	1.00	0.84–1.18	0.96	1,254	1.01	0.83–1.23	0.90	1,426	1.08	0.91–1.28	0.41	1,232	1.17	0.97–1.42	0.10
19 weeks (FL)	1,667	0.89	0.75–1.05	0.18	1,445	0.90	0.75–1.09	0.30	1,655	1.09	0.92–1.29	0.30	1,418	1.16	0.97–1.40	0.11
34 weeks (FL)	1,686	0.86	0.74–1.01	0.07	1,457	0.81	0.69–0.96	0.017	1,671	0.96	0.82–1.12	0.57	1,427	0.98	0.83–1.16	0.85
Birth (CHL)	1,620	0.91	0.77–1.09	0.32	1,425	0.85	0.71–1.03	0.09	1,595	0.92	0.78–1.09	0.34	1,396	0.92	0.77–1.11	0.40
6 months (CHL)	1,324	0.79	0.66–0.94	0.007	1,263	0.78	0.65–0.93	0.006	1,252	0.80	0.67–0.96	0.017	1,212	0.81	0.67–0.97	0.021
12 months (CHL)	1,538	0.88	0.74–1.05	0.16	1,350	0.86	0.72–1.03	0.11	1,591	0.79	0.67–0.94	0.007	1,372	0.82	0.69–0.98	0.028
**Head circumference (SD)**																
11 weeks	1,078	1.04	0.85–1.27	0.69	942	1.07	0.85–1.34	0.56	1,076	1.17	0.96–1.42	0.12	927	1.25	1.00–1.56	0.045
19 weeks	1,665	0.96	0.82–1.14	0.68	1,443	0.98	0.82–1.19	0.86	1,653	1.16	0.98–1.37	0.08	1,416	1.26	1.05–1.51	0.012
34 weeks	1,628	1.02	0.86–1.21	0.80	1,404	1.03	0.85–1.24	0.78	1,614	1.00	0.84–1.18	0.97	1,375	1.06	0.88–1.27	0.55
Birth	1,635	0.89	0.75–1.05	0.15	1,439	0.87	0.73–1.04	0.13	1,609	0.94	0.80–1.11	0.45	1,409	0.95	0.80–1.13	0.56
6 months	1,335	0.88	0.74–1.05	0.16	1,273	0.88	0.73–1.06	0.18	1,263	1.00	0.83–1.20	0.96	1,222	1.01	0.83–1.22	0.94
12 months	1,588	0.89	0.75–1.05	0.16	1,385	0.89	0.75–1.06	0.19	1,644	0.90	0.77–1.06	0.20	1,407	0.92	0.78–1.09	0.35
**Abdominal circumference (SD)**																
11 weeks	999	0.94	0.77–1.16	0.59	875	0.96	0.76–1.21	0.72	1,002	1.16	0.94–1.43	0.16	863	1.21	0.96–1.52	0.10
19 weeks	1,657	0.97	0.82–1.16	0.76	1,436	0.94	0.78–1.14	0.53	1,647	1.10	0.93–1.30	0.28	1,411	1.14	0.95–1.38	0.15
34 weeks	1,687	0.81	0.69–0.96	0.014	1,458	0.78	0.65–0.93	0.006	1,672	0.87	0.74–1.03	0.11	1,428	0.88	0.74–1.05	0.16
Birth	1,633	0.86	0.73–1.02	0.08	1,437	0.83	0.70–1.00	0.045	1,607	0.96	0.81–1.13	0.62	1,407	0.92	0.77–1.10	0.37
6 months	1,340	0.99	0.84–1.17	0.93	1,278	1.01	0.85–1.20	0.90	1,268	1.07	0.90–1.28	0.43	1,227	1.10	0.92–1.31	0.31
12 months	1,580	0.90	0.76–1.05	0.19	1,380	0.87	0.73–1.03	0.11	1,635	1.06	0.90–1.24	0.48	1,402	1.04	0.88–1.23	0.66
**Head: abdominal circumference ratio** *z* **scores (SD)**																
11 weeks	945	1.09	0.88–1.35	0.44	823	1.04	0.83–1.30	0.74	949	1.06	0.86–1.30	0.61	813	1.02	0.81–1.27	0.90
19 weeks	1,654	1.04	0.88–1.22	0.67	1,433	1.09	0.92–1.29	0.34	1,644	1.07	0.91–1.26	0.40	1,408	1.09	0.92–1.29	0.31
34 weeks	1,628	1.32	1.12–1.56	0.001	1,404	1.37	1.15–1.63	0.001	1,614	1.20	1.02–1.41	0.030	1,375	1.22	1.03–1.46	0.025
Birth	1,633	1.13	0.96–1.33	0.15	1,437	1.13	0.95–1.35	0.16	1,607	1.02	0.87–1.20	0.82	1,407	1.07	0.89–1.28	0.47
6 months	1,334	0.96	0.81–1.14	0.66	1,272	0.94	0.79–1.13	0.52	1,262	0.93	0.77–1.11	0.41	1,221	0.91	0.76–1.09	0.32
12 months	1,570	1.07	0.90–1.26	0.45	1,371	1.09	0.92–1.30	0.33	1,625	0.89	0.76–1.05	0.17	1,393	0.91	0.76–1.08	0.28

**Note:**

* Adjusted for maternal BMI, education, smoking, and eczema; and infant sex, gestational age, and breastfeeding duration.

**Abbreviations:** BMI, body mass index; CRL, crown-rump length; FL, femur length; CHL, crown-heel length.

**Table S3:** Growth velocities in relation to eczema at age 6 and 12 months

Growth velocity	Eczema at age 6 months								Eczema at age 12 months							
	Univariate				Multivariate *				Univariate				Multivariate *			
	n	OR	95% CI	*P*-value	n	OR	95% CI	*P*-value	n	OR	95% CI	*P*-value	n	OR	95% CI	*P*-value

**Linear growth (SD)**																
11 weeks	1,003	1.02	0.84–1.24	0.87	973	1.01	0.82–1.25	0.91	1,005	1.15	0.94–1.40	0.18	975	1.22	0.98–1.52	0.07
11–19 weeks	1,003	0.75	0.61–0.93	0.008	973	0.75	0.61–0.93	0.010	1,005	0.94	0.77–1.16	0.58	975	0.94	0.76–1.16	0.55
19–34 weeks	1,003	0.85	0.69–1.04	0.12	973	0.83	0.67–1.02	0.07	1,005	0.99	0.81–1.22	0.96	975	0.98	0.79–1.20	0.81
34 weeks–Birth	1,003	0.95	0.78–1.16	0.61	973	0.93	0.76–1.15	0.51	1,005	0.84	0.69–1.04	0.11	975	0.83	0.68–1.02	0.08
Birth–6 months	1,003	0.81	0.66–0.99	0.040	973	0.82	0.67–1.01	0.07	1,005	0.81	0.66–0.99	0.039	975	0.81	0.66–1.00	0.052
6–12 months	1,003	1.11	0.91–1.36	0.31	973	1.12	0.91–1.38	0.29	1,005	0.91	0.74–1.12	0.36	975	0.90	0.73–1.11	0.34
**Head circumference (SD)**																
11 weeks	748	1.09	0.86–1.38	0.49	726	1.08	0.84–1.40	0.54	749	1.30	1.02–1.66	0.033	727	1.31	1.01–1.70	0.039
11–19 weeks	748	0.85	0.66–1.08	0.19	726	0.82	0.64–1.06	0.14	749	0.95	0.74–1.21	0.66	727	0.94	0.73–1.20	0.63
19–34 weeks	748	1.10	0.87–1.40	0.44	726	1.06	0.83–1.36	0.64	749	0.95	0.75–1.21	0.70	727	0.93	0.72–1.19	0.55
34 weeks–Birth	748	0.84	0.66–1.06	0.15	726	0.85	0.66–1.08	0.19	749	0.88	0.69–1.12	0.29	727	0.88	0.69–1.12	0.30
Birth–6 months	748	1.03	0.81–1.31	0.79	726	1.05	0.82–1.34	0.68	749	1.06	0.83–1.35	0.64	727	1.07	0.84–1.37	0.56
6–12 months	748	0.91	0.72–1.16	0.47	726	0.89	0.69–1.15	0.37	749	0.90	0.71–1.15	0.41	727	0.91	0.70–1.17	0.45
**Abdominal circumference (SD)**																
11 weeks	723	0.97	0.76–1.23	0.78	703	0.94	0.73–1.22	0.66	724	1.24	0.97–1.60	0.09	704	1.24	0.95–1.62	0.11
11–19 weeks	723	0.98	0.78–1.25	0.90	703	0.96	0.76–1.22	0.75	724	0.97	0.76–1.24	0.82	704	0.97	0.76–1.24	0.82
19–34 weeks	723	0.79	0.62–1.00	0.051	703	0.71	0.55–0.92	0.009	724	0.73	0.57–0.93	0.012	704	0.67	0.51–0.88	0.003
34 weeks–Birth	723	1.09	0.86–1.38	0.47	703	1.14	0.89–1.46	0.29	724	1.13	0.88–1.44	0.35	704	1.17	0.91–1.51	0.23
Birth–6 months	723	1.08	0.85–1.37	0.53	703	1.09	0.86–1.40	0.47	724	1.12	0.87–1.44	0.37	704	1.11	0.86–1.43	0.42
6–12 months	723	0.85	0.68–1.08	0.18	703	0.83	0.66–1.05	0.13	724	1.00	0.78–1.27	0.98	704	0.99	0.77–1.26	0.91

**Note:**

* Adjusted for maternal BMI, education, smoking, and eczema; and infant sex, gestational age, and breastfeeding duration.

**Abbreviation:** BMI, body mass index.

## Figures and Tables

**Table 1:** Characteristics of the study population

Characteristics	Total n	%, Median (IQR) or mean (SD)
**Maternal**		
Age at child birth (years)	1,759	31.0 (3.7)
A level or higher, n (%)	1,755	1,111 (63.3)
Smoking in pregnancy, n (%)	1,698	199 (11.7)
Primiparous, n (%)	1,758	917 (52.2)
Eczema in last 12 months, n (%)	1,585	106 (6.7)
Prepregnancy BMI	1,742	24.1 (21.9–27.4)
**Fetal/infant measurements**		
Linear size (mm)		
11 weeks (CRL)	1,489	53.0 (8.9)
19 weeks (FL)	1,728	30.7 (2.1)
34 weeks (FL)	1,747	64.9 (2.7)
Birth (CHL)	1,667	500.7 (18.7)
6 months (CHL)	1,326	675.1 (25.2)
12 months (CHL)	1,592	759.7 (28.7)
Head circumference (mm)		
11 weeks	1,123	69.8 (9.1)
19 weeks	1,726	168.4 (8.6)
34 weeks	1,688	317.8 (10.8)
Birth	1,682	351.0 (12.6)
6 months	1,337	440.7 (14.1)
12 months	1,645	468.6 (14.4)
Abdominal circumference (mm)		
11 weeks	1,044	55.7 (7.5)
19 weeks	1,718	146.3 (9.0)
34 weeks	1,748	307.7 (15.0)
Birth	1,680	317.9 (20.2)
6 months	1,342	477.3 (32.6)
12 months	1,636	497.2 (33.2)
**Infant**		
Male, n (%)	1,759	899 (51.1)
Gestational age at birth (weeks)	1,759	40.1 (39.3–41.0)
Birthweight (kg)	1,750	3.52 (0.47)
Breast feeding (completed months),	1,677	
n (%)		
Never breast fed		270 (16.1)
<1		330 (19.7)
1–3		341 (20.3)
4–6		325 (19.4)
7–11		266 (15.9)
12 or more		145 (8.7)
6-month assessment		
Age (weeks)	1,731	27.4 (26.1–32.9)
Atopic eczema, n (%)	1,698	162 (9.5)
12-month assessment		
Age (weeks)	1,684	53.7 (52.6–55.0)
Atopic eczema, n (%)	1,683	168 (10.0)

**Abbreviations:** BMI, body mass index; CRL, crown-rump length; FL, femur length; CHL, crown-heel length.
