# Supplementary figures and images for: Faltering of prenatal growth precedes the development of atopic eczema in infancy: cohort study
Source: Clin Epidemiol. 2018 Dec 12;10:1851–64. doi: 10.2147/CLEP.S175878 (PMC6296686; doi:10.2147/CLEP.S175878)

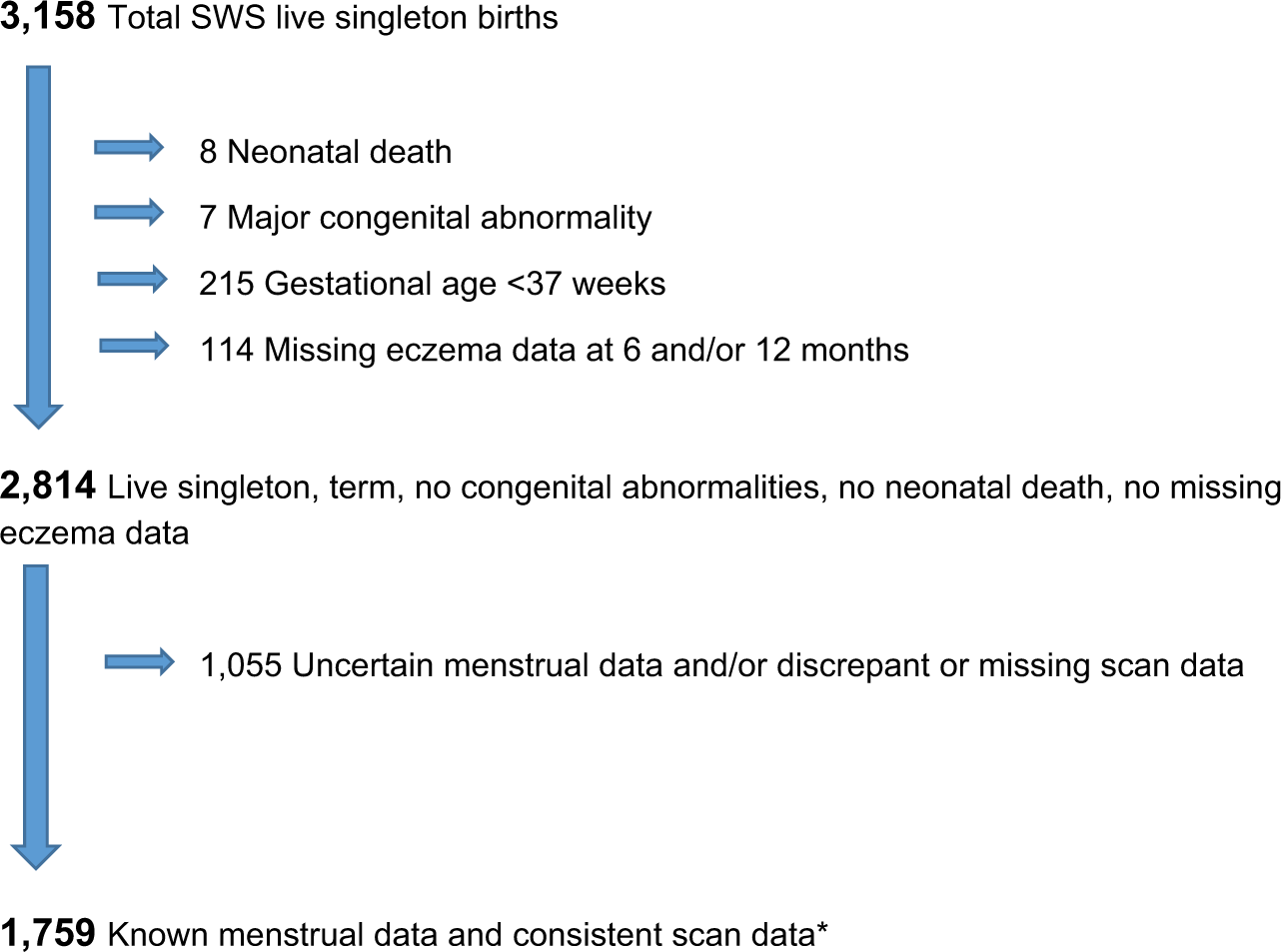

Supplement: Figure S1 — Selection of study group sample from the Southampton Women’s Survey (SWS) cohort. Note: *Non assisted conception, regular cycle, sure/certain of last menstrual period (LMP), not on oral contraceptive pill prior to LMP, dating range scan data available, LMP consistent with date of conception, first positive pregnancy test, scan data, and gestation at birth. [file clep-10-1851s1.tif]

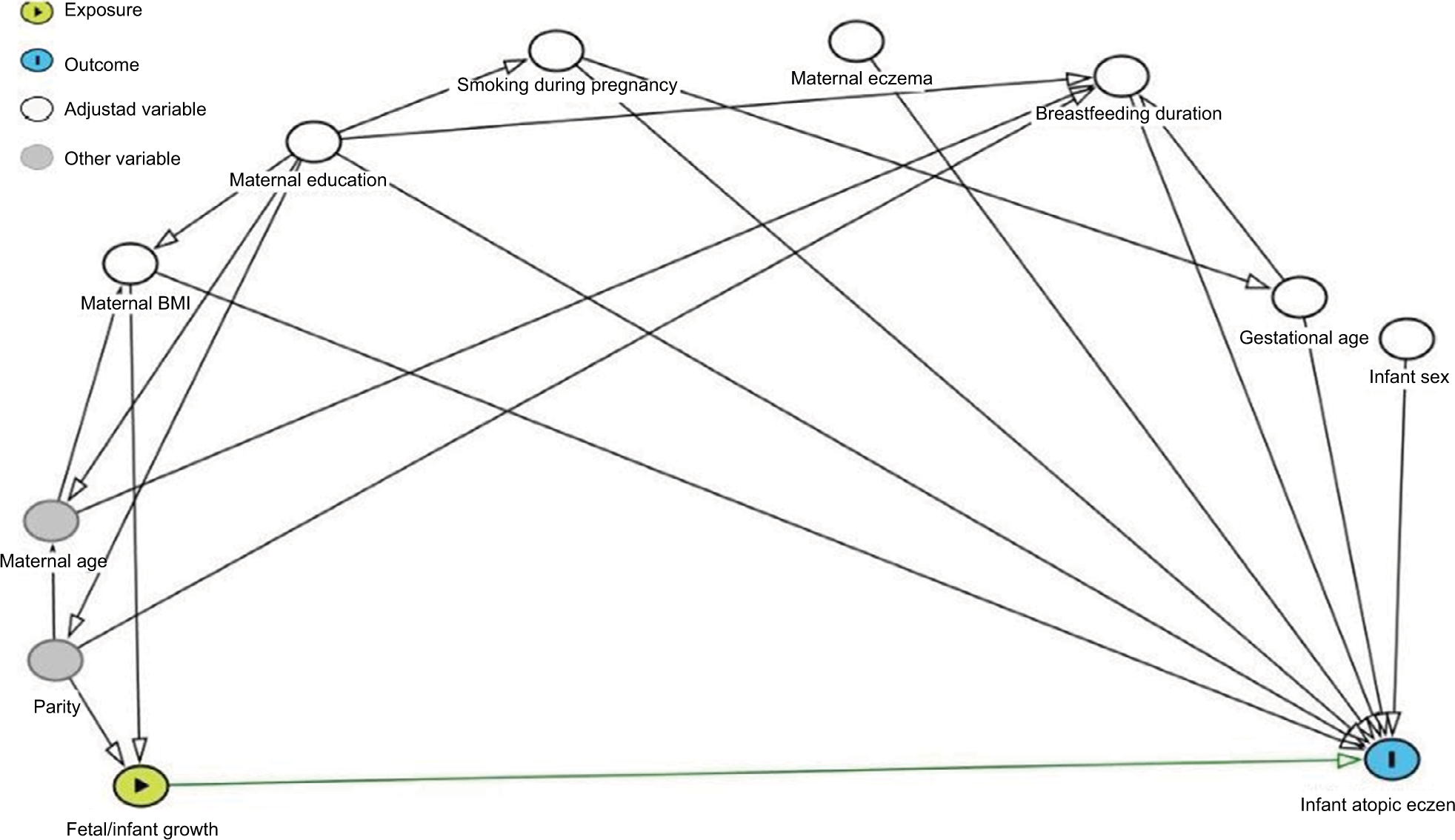

Supplement: Figure S2 — Fetal and infant growth and atopic eczema DAG. Notes: Confounding variables: maternal BMI, maternal education, gestational age, and breastfeeding duration. Competing exposures (variables adjusted for to improve precision of model): maternal eczema, smoking during pregnancy, and infant sex. Abbreviations: BMI, body mass index; DAG, directed acyclic graph. [file clep-10-1851s2.tif]
